# Supplementary material for: Rising rainfall intensity induces spatially divergent hydrological changes within a large river basin
Source: Nat Commun. 2024 Jan 27;15:823. doi: 10.1038/s41467-023-44562-8 (PMC10821892; doi:10.1038/s41467-023-44562-8)
Supplement: Supplementary file 1 — Supplementary Information [file 41467_2023_44562_MOESM1_ESM.pdf]

Supplementary Materials for

Rising rainfall intensity induces spatially divergent hydrological changes within a large river basin

Yiping Wu[1], Xiaowei Yin[1], Guoyi Zhou[2,*], L. Adrian Bruijnzeel[3,4], Aiguo Dai[5], Fan Wang[1], Pierre Gentine[6], Guangchuang Zhang[1], Yanni Song[1], Decheng Zhou[2]

[1] Institute of Global Environmental Change, Department of Earth & Environmental Science, Xi'an Jiaotong University, Xi'an, Shaanxi Province, 710049, P. R. China
[2] Institute of Ecology, School of Applied Meteorology, Nanjing University of Information Science and Technology, Nanjing 210044, P. R. China
[3] Department of Geography, King's College London, London WC2B 4BG, Unite Kingdom
[4] Institute of International Rivers and Eco-Security, Yunnan University, Kunming 650091, P. R. China
[5] Department of Atmospheric and Environmental Sciences, University at Albany, State University of New York, Albany 12222, USA
[6] Department of Earth and Environmental Engineering, Earth Institute, Columbia University, New York 10027, USA
* Corresponding author at: 003054@nuist.ac.cn

This file includes:

Supplementary Figs. 1 to 9
Supplementary Tables 1 to 4

Supplementary Figures

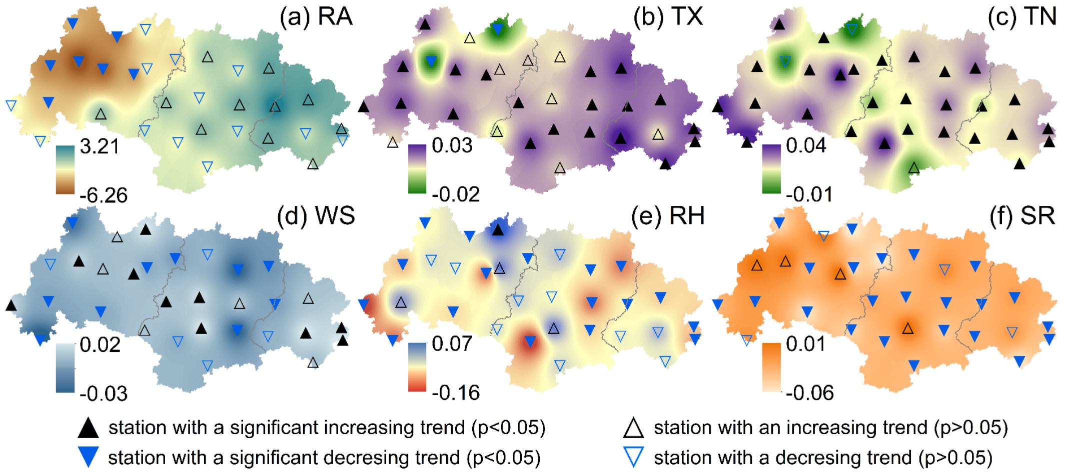

Supplementary Fig. 1 Spatial distribution of annual trends of meteorological factors in the West River Basin over the 54-year (1965–2018) study period. a Rainfall amount (RA; mm). **b** Maximum air temperature (TX; °C). **c** Minimum air temperature (TN; °C). **d** Wind speed (WS; m s^{-1}). **e** Relative humidity (RH). **f** Solar radiation (SR; MJ m^2 d^{-1}). Source data are provided as a Source Data file.

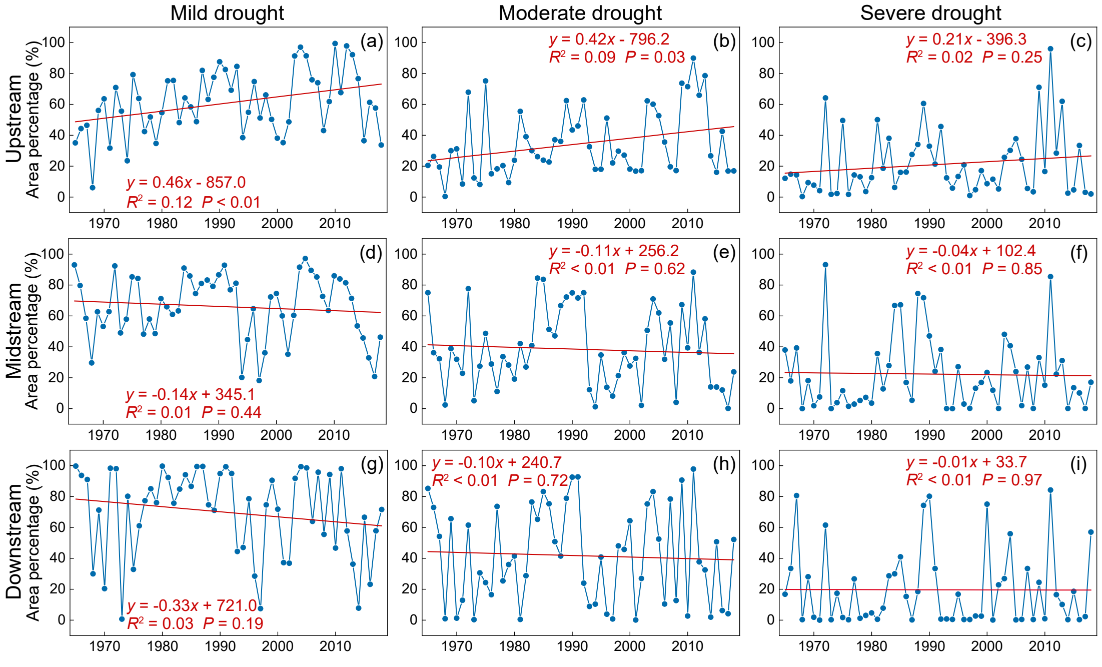

Supplementary Fig. 2 Percentage of area suffering from different grade droughts in the three sub-regions (up-, mid-, and downstream) of the West River Basin from 1965 to 2018. a-c Percentage of area suffering from mild, moderate, and severe drought in the upstream. **d-f** Percentage of area suffering from mild, moderate, and severe drought in the midstream. **g-i** Percentage of area suffering from mild, moderate, and severe drought in the downstream. Source data are provided as a Source Data file.

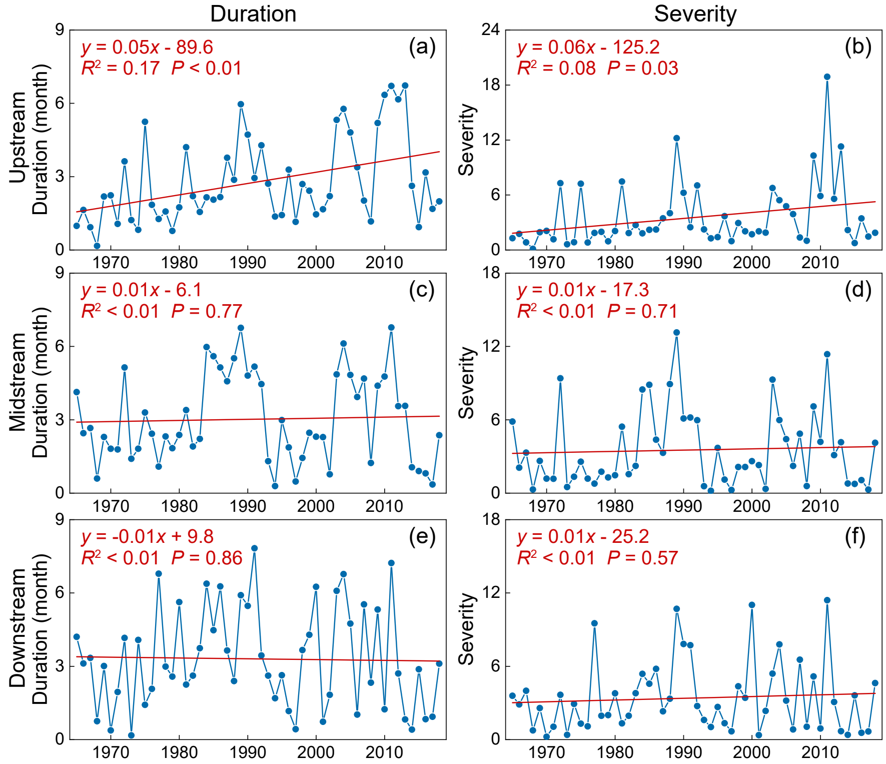

Supplementary Fig. 3 Annual trends of drought duration and severity in the three sub-regions (up-, mid-, and downstream) of the West River Basin from 1965 to 2018. a, b Annual trends of drought duration and severity in the upstream. **c, d** Annual trends of drought duration and severity in the midstream. **e, f** Annual trends of drought duration and severity in the downstream. Source data are provided as a Source Data file.

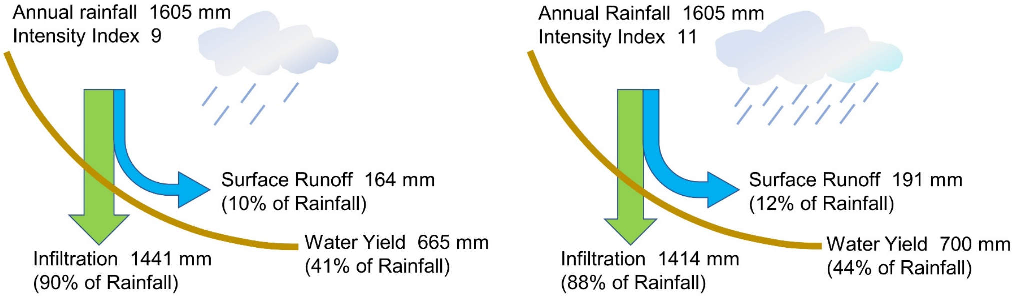

Supplementary Fig. 4 Schematic of SWAT-simulated hydrological responses to rainfall (Wuzhou station) under the same amount of total rain (1605 mm) but at different intensities (RI = 9 for the year 1970 and 11 for the year 2010) for a typical piece of sloping land (slope = 0.5 or 26.6°). Source data are provided as a Source Data file.

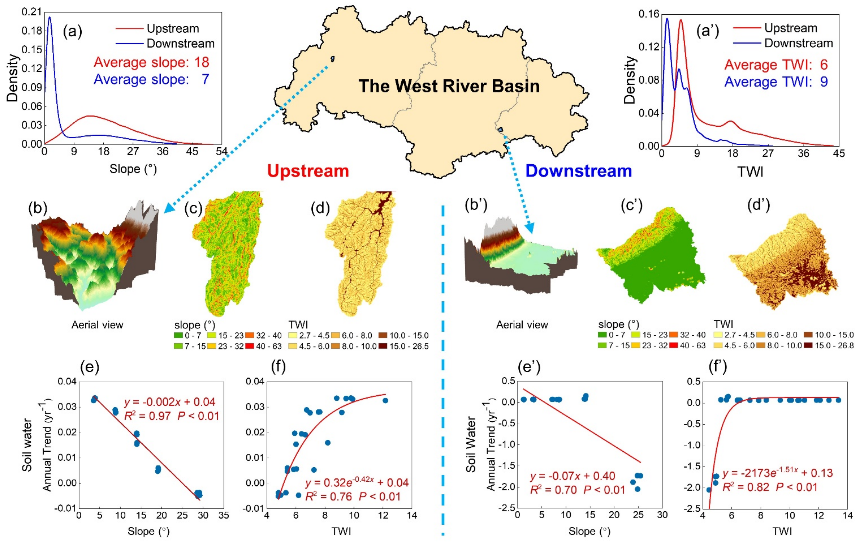

Supplementary Fig. 5 Relationship between soil water trend and slope gradient at the HRU level for a typical small watershed in the up- and downstream part of the West River Basin. **a, b** Density of slope distribution and aerial view of the watershed for a typical small watershed in the upstream. **c, d** Spatial pattern of slope and Topographic Wetness Index (TWI) for a typical small watershed in the upstream. **e, f** Relationship between soil water trend (1965–2018) and slope/TWI for a typical small watershed in the upstream. **a', b'** Density of slope distribution and aerial view of the watershed for a typical small watershed in the downstream. **c', d'** Spatial pattern of slope and TWI for a typical small watershed in the downstream. **e', f'** Relationship between soil water trend and slope/TWI for a typical small watershed in the downstream. Source data are provided as a Source Data file.

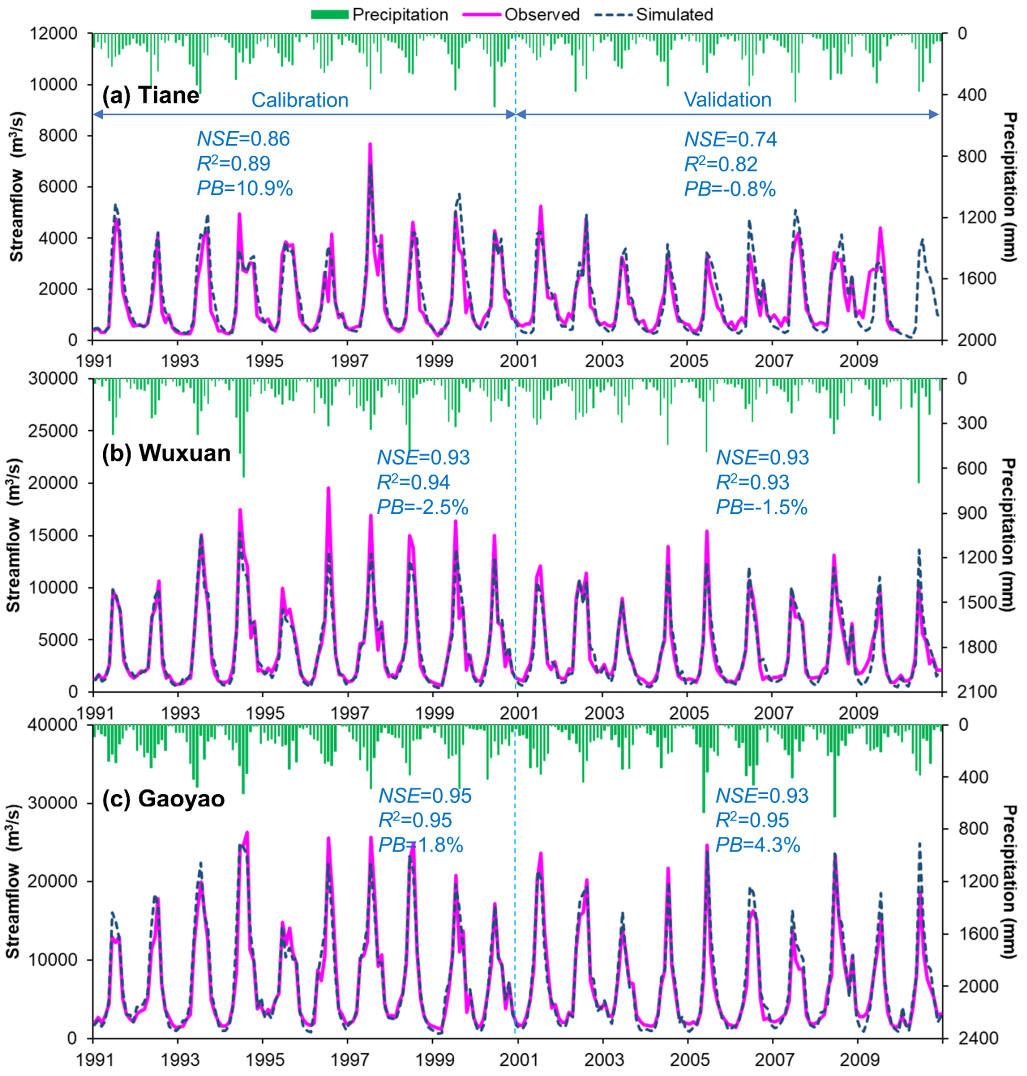

Supplementary Fig. 6 Monthly observed and simulated streamflow at three gauging stations during the ten-year calibration (1991–2000) and validation (2001–2010) periods. a Tiane gauging station (with one year of observations missing during the validation period; 2001-2009). **b** Wuxuan gauging station. **c** Gaoyao gauging station. Source data are provided as a Source Data file.

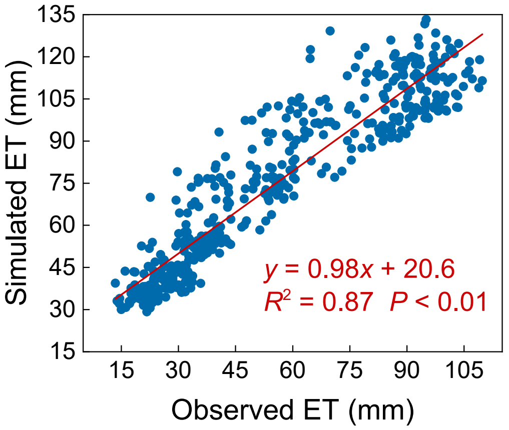

Supplementary Fig. 7 Monthly comparison of SWAT simulated ET and re-analysis ET data based on a fusion of ERA5, MERRA2, and GLDAX2-Noah data during 1980–2017. Source data are provided as a Source Data file.

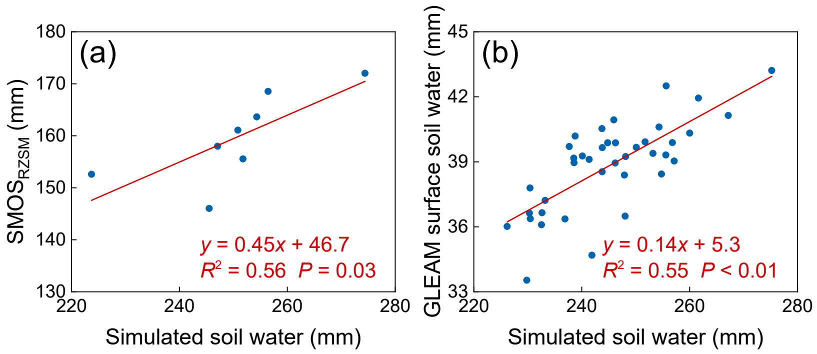

Supplementary Fig. 8 Annual comparison of SWAT-simulated soil water content and two sets of satellite-based soil moisture products. a SWAT-simulated soil water content versus root zone soil water amount from Centre Aval de Traitement des Données SMOS (CATDS) during 2011–2018. **b** SWAT-simulated soil water content versus GLEAM-based topsoil water during 1980–2018. Source data are provided as a Source Data file.

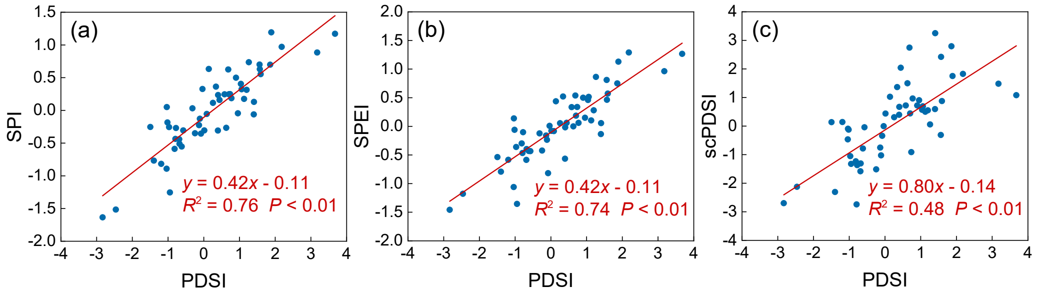

Supplementary Fig. 9 Scatter plots of PDSI versus three other drought indices for the West River Basin from 1965 to 2018. a PDSI versus SPI. **b** PDSI versus SPEI. **c** PDSI versus scPDSI. Source data are provided as a Source Data file.

Supplementary Tables

Supplementary Table 1 Eigenvalues, explained variances, and cumulative explained variances of EOF for PDSI series of the West River Basin during 1965 through 2018.

Mode	Eigenvalue	Explained variance	Cumulative explained variance	Error range	
1	127.1	35.0%	35.0%	113.9	140.2
2	53.1	14.6%	49.6%	47.6	58.5
3	28.6	7.9%	57.5%	25.6	31.6
4	20.0	5.5%	63.0%	17.9	22.0
5	17.8	4.9%	67.9%	16.0	19.7

Note: A significant EOF has an error range that does not overlap with the next higher EOF.

Supplementary Table 2 Comparison of hydrological responses to rainfalls with different intensities.

	Upstream			Downstream		
Scenario	Rainfall 1970	Rainfall 2010	Relative change	Rainfall 1970	Rainfall 2010	Relative change
Rainfall (mm)	825	825	0%	1605	1605	0%
Rainfall intensity index (mm/d)	6.2	6.5	5%	9	10.9	21%
Light-rain (mm)	312	238	-24%	280	264	-6%
Light-rain days	112	96	-14%	125	99	-21%
No-rain days	232	239	3%	187	218	17%
Maximum consecutive dry days	18	22	22%	19	37	95%
Maximum consecutive wet days	13	6	-54%	13	9	-31%
Soil water (mm)	213	212	-0.5%	235	237	0.9%
Water yield (mm)	217	204	-6%	629	692	10%
Surface runoff (mm)	108	100	-7%	415	476	15%
Baseflow (mm)	48	44	-8%	119	123	3%
Maximum streamflow (June-August, m^3/s)	14480	21920	51%	39070	44920	15%
Minimum streamflow (December-February, m^3/s)	261	255	-2%	762	952	25%

Supplementary Table 3 Calibrated values of the five key parameters of SWAT for the West River Basin.

Parameter	Description	Adjustment range	Calibrated value/change		
			Up	Mid	Down
CN2	Curve number II value	–10% ~ 10%	0.1%*	-5.6%*	2.5%*
ALPHA_BF	Baseflow recession factor	0.001 ~ 0.1	0.061	0.099	0.060
ESCO	Soil evaporation compensation factor	0.01 ~ 1.0	0.710	0.901	0.292
SOL_AWC	Soil available water capacity	–10% ~ 10%	3.4%*	7.5%*	4.0%*
CH_K2	Effective hydraulic conductivity in main channel (mm h^{-1})	–15 ~ 15	+13.96$^{\$}$	+8.86$^{\$}$	+2.50$^{\$}$

* refers to a relative change; $^{\$}$ value added to existing parameter value.

Supplementary Table 4 Classification of drought grades based on PDSI.

Grade	PDSI	SPI or SPEI
Extreme	< –4.0	< –2.0
Severe	–3.99 ~ –3.0	–1.99 ~ –1.5
Moderate	–2.99 ~ –2.0	–1.49 ~ –1.0
Mild	–1.99 ~ –1.0	–0.99 ~ –0.5
No	–0.99 ~ 0.99	–0.49 ~ 0.49
Wet	>1.0	>0.5
